# Magnitude of dental caries, missing and filled teeth in Malawi: National Oral Health Survey

**DOI:** 10.1186/s12903-016-0190-3

**Published:** 2016-03-09

**Authors:** Kelias Phiri Msyamboza, Enock Phale, Jessie Mlotha Namalika, Younam Mwase, Gian Carlo Samonte, Doubt Kajirime, Sewedi Sumani, Pax D. Chalila, Rennie Potani, George Chithope- Mwale, Damson Kathyola, Weston Mukiwa

**Affiliations:** World Health Organization, Malawi Country Office, Lilongwe, Malawi; University of Malawi, College of Medicine, Blantyre, Malawi; Ministry of Health Headquarters, Lilongwe, Malawi; Ministry of Health, Kamuzu Central Hospital, Lilongwe, Malawi; Malawi College of Health Services, Lilongwe, Malawi; Ministry of Health, Queen Elizabeth Central Hospital, Blantyre, Malawi; Ministry of Health, Bwaila Hospital, Lilongwe, Malawi; Ministry of Health, Zomba Central Hospital, Zomba, Malawi; Ministry of Health, Research Unit, Lilongwe, Malawi; Dental Association of Malawi, Lilongwe, Malawi; WHO Malawi, ADL House, City Centre, P.O. Box 30390, Lilongwe, 3 Malawi

**Keywords:** Oral health, Dental caries, Non-communicable diseases, Sub-Saharan Africa, Malawi

## Abstract

**Background:**

Oral health problems are significant cause of morbidity particularly in sub-Saharan Africa. In Malawi, routine health management information system data over the years showed that oral health problems were one of the top ten reasons for outpatient attendance. However, to date, no national oral survey has been carried out to determine the prevalence of oral health problems.

**Methods:**

A national population-based cross-sectional survey was conducted in 2013. A total of 130 enumeration areas (EAs) were randomly selected and from each EA, 40 participants were randomly selected as per WHO STEPS survey protocol. Eligible participants were 12, 15, 35–44 and 65–74 year old. A multi-stage sampling design was used to obtain a national representative sample of these age groups. Oral examination was based on WHO diagnostic criteria (2010).

**Results:**

A total of 5400 participants were enrolled in the survey. Of these: 3304 (61.3 %) were females, 2090 (38.7 %) were males; 327 (6.9 %) were from urban and 4386 (93.1 %) from rural areas; 1115 (20.6 %), 993 (17.3 %), 2306 (42.7 %) and 683 (12.6 %) were aged 12, 15, 35–44, 65–74 years respectively. Among 12 year-old, 15 year-old, 35–44 and 65–74 year age groups, prevalence of dental caries was 19.1, 21.9, 49.0 and 49.2 % respectively, overall 37.4 %. Prevalence of missing teeth was 2.7, 5.2, 47.7 and 79.9 %, overall 35.2 %. Prevalence of filled teeth was 0.2 %, 1.3 %, 8.7 %, 12.7 %, overall 6.5 %. Prevalence of bleeding gums was 13.0, 11.8, 30.8 and 36.1 %, overall 23.5 %. Toothache, dental caries and missing teeth were more common in females than males; 46.5 % vs 37.9 %, 40.5 % vs 32.4 %, 37.7 % vs 30.1 % respectively, all *p* < 0.05. Prevalence of dental caries and missing teeth in urban areas were as high as in the rural areas; 33.3 % vs 37.4 % and 30.9 % vs 33.7 % respectively, all *p* > 0.05. The mean number of decayed, missing and filled teeth (DMFT) in 12, 15, 35–44, 65–74 year old was 0.67, 0.71, 3.11 and 6.87 respectively. Self- reported brushing of teeth was poor with only 35.2 % of people brushed their teeth twice a day and tobacco smoking was high, particularly among adult males where one in five (22.9 %) was a smoker.

**Conclusion:**

This study demonstrated that oral health problems are major public health problems in Malawi. One in five (21 %) adolescents aged 12–15 years and half (49 %) of adults aged 35 years or more had dental caries, half (48 %) and 80 % of the population aged 35–44, 65–74 years had missing teeth respectively. Toothache, dental caries and missing teeth were more prevalent in females than males and prevalence in urban was as high as in rural areas. Oral hygiene was poor with less than 40 % of the population brush their teeth twice a day and tobacco smoking was high, particularly in men where prevalence was 23 %. These findings could be used to develop evidence-informed national policy, action and resource mobilization plan and community based interventions to reduce the prevalence of oral health problems in Malawi.

## Background

Dental caries is a major oral health problem affecting 60–90 % of school age children and majority of adults. Although the disease level is relatively low in Africa compared to Asian and Latin American countries, it is expected that the incidence will increase in many developing countries in Africa because of growing consumption of sugar, inadequate exposure to fluorides and limited access to oral health services [1, 2].

In Malawi, there is lack of population-based data on the status of oral health problems. However, hospital based data from health management information system (HMIS) suggest that oral health problems are the sixth commonest cause (after malaria, upper respiratory conditions, musculoskeletal pain, diarrhoea and pneumonia) for outpatient department (OPD) attendance. In 2010, of the 1,726,065 OPD visits, 57,234 (3.3 %) were due to oral health problems [3]. The main aim of this study was to determine the burden of oral health problems in Malawi and use the data to develop evidence-informed oral health policy, strategic plan, interventions and mobilize resources.

## Methods

### Survey design

This was a national population- based cross-sectional survey. WHO STEPwise approach for assessing risk factors for chronic non-communicable diseases including oral health problems was used. STEPS is a sequential process starting with gathering information on socio-demographic and risk factors (age, sex, education, occupation, tobacco and alcohol use etc) using semi-structured questionnaire (Step 1), then moving to physical oral examination (Step 2). STEP 3 which involves laboratory tests was not applicable [4, 5].

### Data collection, tools and eligible participants

A workshop was held to adapt WHO Oral Health Survey questionnaire, child and adult examination forms [6]. Based on WHO Oral Health Survey methods and in line with oral health indicators, the following age groups were eligible for the survey, 12, 15, 35–44 and 65–74 years old. The tools were pre-tested and finalized. Inter-examiner reliability exercise was conducted by experts to assess the consistency of the examiners and there was high agreement.

### Sample size

Sample size was estimated using the formula: n = Z^2^P(1 − P) /e^2^

Where *n* = sample size, Z = level of confidence, P = baseline prevalence of the condition and e = margin of error. Given the estimated prevalence, *P* = 0.50, *Z* = 1.96 (at 95 % confidence interval), *e* = 0.05. The sample size was adjusted for design effect for complex sample design set at 1.5, age-sex estimates in the 12, 15, 35–44 and 65–74 age groups range and a non-response rate of 10 %. The minimum calculated sample size was therefore multiplied by 1.5 and by 8, and then divided by 0.9 to adjust for design effect, age-sex estimates and non-response rate respectively. With these adjustments, the final sample was 5120.

### Study design

This survey was designed to obtain data that would be a representation of the whole Malawi. To achieve this, a multi-stage sampling method was used to select enumeration areas (EAs), households and eligible participants at each of the selected households (three stages).

### Selection of enumeration areas

Administratively, Malawi is divided into 28 districts. Each district is sub-divided into administrative units (traditional authorities). Each administrative unit is sub-divided into enumeration areas (EAs) by the National Statistical Office (NSO). Enumeration areas are classified as urban or rural. Each EA has a sketch map drawn by NSO. The sketch map shows the EA boundaries, location of buildings, and other landmarks. List of EAs from NSO served as the sampling frame for the oral health Survey. In accordance with WHO STEPS Manual Part 2 Section 2, the recommended number of participants to be selected at each primary sampling unit (PSU) is 30–50 [7]. For this survey, it was proposed that we recruit 40 participants in each enumeration area. Given the estimated required sample size of 5120, the total number of EAs selected were therefore 5120/40 = 128. Thus a total of 128 EAs were randomly selected from the list of all EAs in Malawi. Probability Proportional to Size (PPS) sampling method was used to randomly sample the 128 enumeration areas from the sampling frame.

The PPS sampling method was as follows: The first step was sorting the EAs (in Microsoft Excel®) in descending order of population (largest to the smallest). Total population of all EAs in Malawi was then calculated. Thirdly, a column of cumulative total population of EAs was created. Sampling interval was calculated by dividing total population by 128 (total number of EAs to be selected). Random number (the seed) was generated by computer in the excel using the formula RANDBETWEEN(sampling interval). The EAs whose cumulative total contain the seed was the first to be selected. The 2^nd^, 3^rd^, …….., 128^th^ EA was selected by systematically adding the random number to the sampling interval.

### Selection of households and eligible participants

To calculate sampling interval for each EA, the total number of households in the EA was divided by 40 (number of households to be selected). Households in the EA were randomly selected using systematic sampling methods. EA maps from NSO contain names of villages or township of the given EA. Participants aged 12, 15, 35–44 and 65–74 years were eligible. In each age group, only one participant per household was selected using simple random method.

### Conduct of the survey

#### National Task Force

The national task force was formed. It consisted of representatives from Department of Clinical Services (MoH), Health Education Unit (MoH), Health Management Information System (MoH) and Environmental Health (MoH), Kamuzu Central Hospital (MoH), National Statistical Office and World Health Organisation. The members of the national team drafted the proposal and adapted survey tools, and conducted the training of the data collectors. Department of Clinical Services (MoH) coordinated all the survey activities.

#### Field Teams

There were ten field survey teams. Each team comprised of eight members with the following composition: one supervisor (planning and checking the completeness of questionnaires), two enumerators (questionnaire), two dental clinical officers (oral health examination), one Health Surveillance Assistant (notifying the village heads of the selected villages about the survey, date and time of the survey time), a village guider (to introduce the team to households) and a driver (transport). A total of 80 (10×8) surveyors were therefore involved in the survey for an estimated period of 20 days including training.

### Data management

Data were entered in Epi Info 7 version 3.5.4 (Center for Disease Control, Atlanta, GA, USA) and exported to SPPS for Windows version 20 (Chicago, IL, USA) for analysis. Chi-square test was used to evaluate the differences in prevalence and student’s *t*-test or Mann–Whitney for differences in mean for caries, missing and filled teeth (as dependent variables) by age, sex, residential area (as independent variables). Multivariate logistic regression modeling was used to ensure allowance for potential confounding variables.

### Ethical consideration

Ethical approval was granted by National Health Science Research Committee. Informed written consent was obtained from the individual adult participants. For children aged 12–15 years, informed written consent was obtained from their parents or guardians.

## Results

### Socio-demographic characteristics of participants enrolled in the survey

A total of 5400 participants were enrolled in the survey. Of these, 3304 (61.2 %) were females and 2090 (38.8 %) were males, 20.6, 42.7 and 12.6 % were aged 12, 35–44 and 65–74 years respectively, 6.9 % were from urban and 93.1 % were from urban areas. Of the 2517 adults aged 25–74 years, 9.7 and 15.2 % were tobacco smokers and alcohol drinkers respectively. Tobacco smoking and alcohol drinking were more common in males than females, 22.9 % vs 3.1 %, 35.0 % vs 5.4 % respectively, *p* < 0.5 (Table 1).

**Table 1:** Socio-demographic characteristics of participants enrolled in the Malawi National Oral Health Survey 2013

	Males		Females		Both sexes	
	n	%	n	%	n	%
Age group years):						
12	541	25.9	574	17.4	1,115	20.6
15	397	19.0	536	16.2	933	17.3
35–44	738	35.3	1,568	47.5	2,306	42.7
65–74	251	12.0	432	13.1	683	12.6
Others	170	7.8	194	5.6	363	6.7
All	2,090	100.0	3,304	100.0	5,400	100.0
Location:						
Urban	167	9.1	160	5.6	327	6.9
Rural	1,666	90.9	2,720	94.4	4,386	93.1
All with known location	1,883	100.0	2,880	100.0	4,713	100.0
Tobacco use in adults aged 25–64 years	831	22.9*	1,686	3.1	2,517	9.7
Alcohol use in adults aged 25–64 years	831	35.0*	1,686	5.4	2,517	15.2

*N* = number participants in the group, *%* = percentage, *statistically significant, males vs females, *p* = <0.05

### Self-reported brushing of teeth and selected oral health problems

Of the 5400 participants, 39.8 % said they cleaned their teeth three times a day, 35.2 % said twice, 19.7 % said once a day and 2.9 % said they never cleaned their teeth. Use of fluoridated toothpaste was reported by 42.6 % of the participants. Reported prevalence of toothache and gum bleeding in the past 12 months prior to the survey was 43.1 and 19.8 % respectively. Toothache was more common in females than males, 46.5 % vs 37.9 %, *p* < 0.05 (Table 2).

**Table 2:** Self-reported brushing of teeth and selected oral health problems experienced in the last 12 months prior to the survey

	Males (*n* = 2,090)	Females (*n* = 3,304)	Both sexes (*N* = 5,400)
How often people clean their teeth:			
%Never	3.2	2.9	2.9
%Once a day	24.2	16.8	19.7
%Twice a day	36.5	34.4	35.2
%Three times or more a day	32.5	44.4	39.8
%Use of fluoridated toothpaste	45.0	41.2	42.6
%Experienced toothache in the last 12 months prior to the survey	37.9	46.5*	43.1
%Experienced bleeding gums in the last 12 months prior to the survey	19.9	19.7	19.8

*%* percentage, *statistically significant, males vs females, *p =* <0.05

### Prevalence of decay, missing and filled teeth (DMFT)

Detailed information of the prevalence and mean of decayed, missing and filled teeth are shown in Table 3. Among 12 year-olds, 15 year-olds, 35–44 and 65–74 year age group, the prevalence of caries were 19.1, 21.9, 49.0 and 49.2 %% respectively. Dental caries were more common in females than males, 40.5 % vs 32.4 %, *p* < 0.05. Prevalence of dental caries was statistically similar in urban and rural, 33.3 % vs 37.4 %, *p* > 0.05.

**Table 3:** Prevalence and mean number of decayed, missing and filled teeth by age, gender and location: Malawi national oral health survey 2013

	N	Prevalence of decayed, missing and filled teeth						Mean number of decayed, missing and filled teeth (DMFT)							
		Decayed teeth		Missing teeth		Filled teeth		Decayed teeth		Missing teeth		Filled teeth		DMFT	
		%	95 % CI	%	95 % CI	%	95 % CI	No. of teeth affected	mean	no. of teeth affected	mean	no. of teeth affected	mean	no. of teeth affected	mean
Age (years):															
12	1,114	19.1	16.8–21.4	2.7	1.7–3.7	0.2	−0.1–0.5	459	0.41	285	0.26	4	0.00	748	0.67
15	933	21.9	19.2–24.6	5.2	3.8–6.6	1.3	0.6–2.0	459	0.49	195	0.21	13	0.01	667	0.71
35–44	2,305	49.0	47.0–51.0	47.7	45.7–49.7	8.7	7.5–9.9	3,278	1.42	3,636	1.58	264	0.11	7,178	3.11
65–74	683	49.2	45.5–52.9	79.9	76.9–82.9	12.7	10.2–15.2	964	1.41	3,506	5.13	232	0.34	4,702	6.87
Others	356	37.4	32.4–42.4	41.3	36.2–46.4	13.1	9.6–16.6	423	1.17	679	1.87	86	0.24	1,188	3.27
All	5,391	37.4	36.1–38.7	35.2	33.9–36.5	6.5	5.8–7.2	5,583	1.03	8,301	1.54	599	0.11	14,483	2.68
Gender:															
Male	2,090	32.4	30.4–34.4	30.1	28.1–32.1	6.6	5.5–7.7	1,668	0.80	3,069	1.47	200	0.10	4,937	2.36
Female	3,301	40.5*	38.8–42.2	37.7*	36.0–39.4	6.6	5.8–7.4	3,846	1.17	5,271	1.60	401	0.12	9,518	2.88
Both sexes	5,391	37.4	36.1–38.7	34.8	33.5–36.1	6.5	5.8–7.2	5,514	1.02	8,340	1.55	601	0.11	14,455	2.68
Location:															
Urban	327	33.3	28.2–38.4	30.9	25.9–35.9	5.2	2.8–7.6	239	0.73	377	1.15	40	0.12	656	2.01
Rural	4,384	37.4	36.0–38.8	33.7	32.3–35.1	7.2	6.4–8.0	4,612	1.05	6,496	1.48	535	0.12	11,643	2.66

*CI* Confidence interval, *DMFT* decayed, missing, filled teeth, *N* number of participants in the group, *no.* number, *%* percentage, *statistically significant, prevalence in males vs females, urban vs rural, *p* < 0.05. The mean number of decayed, missing and filled teeth was calculated as the number of affected teeth divided by the total number (N) of participants in the group

Prevalence of missing teeth due caries or other reasons was 2.7, 5.2, 47.7 and 79.9 % in 12, 15, 35–44, and 65–74 year olds respectively. More females than males had missing teeth, 37.7 % vs 30.1 %, p < 0.05. Prevalence of missing teeth was statistically similar in urban and rural areas, 30.9 % vs 33.7 %

Prevalence of filled teeth with or without caries was about 1 % in 12 and 15 year olds and 11 % in those aged 35 years or more. The mean number of decayed, missing and filled teeth (DMFT) in 12, 15, 35–44, 65–74 year olds was 0.67, 0.71, 3.11 and 6.87 respectively.

## Discussion

In sub-Saharan Africa, recent studies on the prevalence oral health problems are limited, particularly in adults, and prevention and care is often neglected. The World Health Organization (WHO) Africa Region Office has recommended more commitment for oral health at the country level [8, 9]. This study was the first national population-based oral health survey on different age groups in Malawi and east and southern Africa. The study demonstrated that oral health problems are major public health problems with over 43 % of participants reported that had toothache in the last 12 months prior to the survey, one in five (20.3 %) children aged 12–15 years and about half (49.1 %) of the adults aged 35 years or more had caries, 47.8 and 79.9 % of adults aged 35–44, 65–74 had missing teeth and one in three (33.4 %) had gum disease respectively. This information could be used to develop or update evidence- informed national oral health strategic and resource mobilization plan.

The findings of this study were consistent with the findings from other studies that prevalence of dental caries is relatively low in low-income compared to high- income countries. In sub-Saharan Africa, prevalence of dental carries in children and adolescents have been estimated to range from 10 to 60 %, while in adults, the estimated prevalence is about 57 % [10–14]. This study estimated that the prevalence of dental caries was 20.3 % in children and 49.1 % in adults.

The other lesson learnt from this study was that in terms of risk factors; gender was an important risk factor. Prevalence of toothache, dental caries and missing teeth were significantly more common in females than males; 46.5 % vs 37.9 %, 40.5 % vs 37.4 %, 37.7 % vs 30.1 % respectively. However, location (urban or rural) was not a risk factor in this population. The estimated high prevalence of tobacco smoking in men (23 %) than in women (3 %) were similar to the findings of STEPS survey conducted in 2009 (25 and 3 % respectively) [15, 16]. The prevalence of dental caries, missing teeth and gum disease were as high in urban as in rural areas; 33.3 % vs 37.4 %, 30.9 % vs 33.7 % and 23.1 % vs 23.2 % respectively. This would suggest that in Malawi, population based interventions for prevention and control of oral health problems should target both urban and rural areas.

Other striking findings from this study were poor oral hygiene with only 35 % of the people reported cleaning their teeth at least twice a day and less than half (43 %) of these used fluoridated toothpaste. This study also showed that there was low number of people with filled teeth which was present in only 10 % of adults aged 35 or more. This would suggest inadequate availability or lack of restorative or preventive dental care and treatment was limited to pain relief or emergency care by tooth extraction as reported by other studies [17].

### Limitations of the study

In the sample of 5400 participants, there was over-representation (61 %) of females compared to males (39 %) and rural (93 %) compared to urban population (7 %). Males were away working in the field during the time of the study. Malawi is largely rural according to data from National Statistical Office with 85 % of population living in rural areas. Our study followed a similar pattern. It is uncertain whether over-representation of women and rural population had influence on the results. Nonetheless, this national population based oral health survey was the first in Malawi and one of the few studies that targeted multiple age groups with a larger sample size of over 5000 people.

## Conclusion

This study demonstrated that oral health problems are major public health problems in Malawi. One in five (21 %) adolescents aged 12, 15 years old and half (49 %) of adults aged 35 years or more had dental caries, half (48 %) and 80 % of the population aged 35–44, 65–74 years had missing teeth respectively. Toothache, dental caries and missing teeth were more frequent in females than males and prevalence in the urban was as high as in rural areas. Oral hygiene was poor with less than 40 % of the population brushed their teeth twice a day and tobacco smoking was high, particularly in men where prevalence was 23 %. These findings could be used to develop evidence-informed national policy, action and resource mobilization plan and community based interventions to reduce the prevalence oral health problems in Malawi.
